# COVID-19's Effect on Pregnancy Care for Incarcerated People

**DOI:** 10.1089/heq.2022.0035

**Published:** 2022-06-10

**Authors:** Camille Kramer, Ashley-Devon Williamston, Rebecca J. Shlafer, Carolyn B. Sufrin

**Affiliations:** ^1^ Department of Gynecology and Obstetrics, Johns Hopkins University School of Medicine, Baltimore, Maryland, USA.; ^2^ Department of Pediatrics, University of Minnesota Medical School, Minneapolis, Minnesota, USA.; ^3^ Department of Health, Behavior and Society, Johns Hopkins Bloomberg School of Public Health, Baltimore, Maryland, USA.

**Keywords:** COVID-19, incarceration, pregnancy, pregnancy care, prisons, jails

## Abstract

**Objective/background:**

Prisons and jails are high risk environments for COVID-19. Little is known about COVID-19's impact on pregnancy care for the tens of thousands of pregnant people who pass through these institutions each year. This study aimed to describe how COVID-19 has influenced prisons' and jails' pregnancy care services.

**Methods:**

We conducted a cross-sectional survey of a convenience sample of U.S. prisons and jails and report descriptive statistics.

**Results:**

We received 17 responses. Sites reported changes in prenatal care delivery, support programming, housing, and visitation. Most sites implemented changes in housing arrangements to quarantine individuals at-risk for COVID-19. Many sites increased their use of virtual technology to supplement for suspended in-person appointments, programming, and visitation.

**Conclusions:**

The impact of COVID-19 on pregnancy care delivery and support services for incarcerated pregnant people is variable. As the pandemic continues, research and policy should ensure that incarcerated pregnant people have access to full scope pregnancy care.

## Introduction

In April, 2020, Andrea Circle Bear, a 30-year-old Lakota woman serving a 26-month sentence in federal prison, acquired COVID-19 and died after giving birth on a ventilator.^1^ The Bureau of Prisons later revealed that she had pre-existing conditions, which likely exacerbated the effects of the virus. Her death corroborated concerns voiced by many that individuals with unique health care needs, including pregnancy, have an increased risk of catching and dying from COVID-19 in custody.^2^

It is well established that United States (U.S.) prisons and jails are high-risk environments for COVID-19 transmission.^3^ Crowded conditions, workers moving in and out daily, and insufficient personal protective equipment and hygiene supplies have led carceral institutions to be infection hotspots. By June 2020, the COVID case rate for incarcerated individuals was 5.5 times higher than the U.S. population case rate per 100,000 individuals, and by February 2022, there were over 576,000 COVID cases among imprisoned people.^4,5^ Many institutions have had to restructure their health care delivery to respond to COVID-19.

Despite the national dialog regarding COVID-19 and incarcerated individuals, the needs of pregnant incarcerated people have received little attention.^6,7^ Yet they are at a higher risk of severe illness than nonpregnant individuals,^8^ and there are about 58,000 admissions of pregnant people to prisons and jails each year.^9,10^ COVID-imposed constraints on health care service delivery in carceral facilities likely affect care for pregnant people in custody, who have time-sensitive and frequent health care needs, including abortion, prenatal, birth, and postpartum, and mental health care.

Although nearly 2 years have passed since the death of Andrea Circle Bear, COVID-19 is still a threat to the safety and well-being of incarcerated, pregnant individuals, and there is still a gap in information about health care for pregnant women in carceral facilities. This study aims to understand how pre-COVID policies and practices for pregnant people in U.S. carceral facilities have been impacted and changed because of COVID-19.

## Materials and Methods

We conducted a cross-sectional survey of a convenience sample of state prisons and jails from July to September 2020 to obtain data about their pregnancy policies and practices before and during the COVID-19 pandemic. We recruited state prisons and jails from a previous study, the Pregnancy and Prison Statistics (PIPS) study, a national study of pregnancy outcomes and policies conducted at 22 state prison systems and 6 county jails.^9,10^ We identified additional sites by searching state Department of Corrections (DOC) websites to gather contact emails and utilized snowball sampling. We sent the survey to 47 prisons and 11 jails. Only sites that housed pregnant people were eligible for participation.

We adapted the survey from the PIPS baseline questionnaire about pregnancy services, adding questions assessing changes in the logistics and availability of pregnancy care, support programs (e.g., doulas, parenting classes), housing and quarantine protocols, visitation, and arrest/release practices.^9,10^ We sent an initial recruitment email (and two subsequent reminder emails) explaining the study with a link to the survey. We requested the survey be completed by someone with knowledge of the facility's health care policies and practices for pregnant people, and asked whether they had a medical or custody role.

The survey was administered electronically using REDCap, a secure web-based survey and data collection platform.^11^ Data were analyzed for descriptive statistics. Proportions were calculated based on the number who responded to each question. Due to the small size of the sample and known variability in health care among carceral facilities, no test of association was performed. This study was approved by the Johns Hopkins School of Medicine Institutional Review Board.

## Results

Twenty-five surveys were returned; seven individuals did not complete any outcome of interest, and we excluded one duplicate response. Thus, there were 17 analyzable surveys, 13 from prisons and 4 from jails (analyzable response rate 28%). Reporting sites represented a geographic range and had a variety of health care service delivery models, and the majority of respondents had a health care role and decision-making capacity at the facility (Table 1).

**Table 1.** Characteristics of Facilities and Respondents to Survey About Prison and Jail Changes to Pregnancy Care During COVID-19 (***n*=17**)

Characteristic	***n*** (%)
Facility type	
Prison	13 (76)
Jail	4 (24)
Region^a^	
West	2 (14)
Midwest	4 (29)
South	5 (36)
Northeast	2 (14)
Role of person completing survey	
Custody role^b^	2 (15)
Health care role^b^	12 (92)
Decision-making role	10 (77)
Nondecision-making role	3 (23)
Health care services delivery^c^	
Directly through facility	5 (29)
Private contract	4 (24)
Community nonprofit	4 (24)
Clinic or hospital contract	3 (18)
University health center contract	1 (6)
Individual health care provider	2 (12)

^a^ Some responses had missing data. Proportions were calculated based on the number who responded to the question.

^b^ One individual reported both a custody and health care role.

^c^ Facilities could indicate multiple service delivery models.

Table 2 reports details of pre-pandemic services and subsequent changes during the pandemic. One site shifted pregnancy care to a medical prison because their contracted prenatal care provider was no longer allowed to enter the facility. Sites that had off-site care for consultations or acute pregnancy concerns retained those arrangements. Concomitantly, several sites increased their use of telehealth. Five prisons and all jails required people to pay a co-pay for “sick call” visits pre-COVID, and some sites lifted this during the pandemic. All facilities suspended in-person visitation (Table 2). This also extended to pregnancy and postpartum support services and programming, with some canceling these altogether, while others moved to virtual format. Other changes included offering free phone calls (*n*=8, 62%) or video conferencing (*n*=6, 46%) between incarcerated people and their families.

**Table 2.** Prison (***n*=13) and Jail (*n*=4) Changes to Pregnancy Care and Other Practices Due to COVID-19**

Practice/standard of care	Sites providing service pre-COVID-19 ***n*** (%)^a^		Sites with change to service due to COVID-19 ***n*** (%)^b^		Information about changes
	Prisons	Jails	Prisons	Jails	
On-site routine prenatal care	10 (77)	4 (100)	1 (8)	0	One prison changed from on-site prenatal care to off-site prenatal care at a prison medical facility in their state
Off-site routine prenatal care	9 (69)	4 (100)	0 (0)	0	Before COVID, seven prisons and all jails provided on-site and off-site routine prenatal care and two prisons provided only off-site routine prenatal care. There was no change.
On-site evaluation of acute pregnancy problems	8 (67)	3 (75)	0	0	One jail and four prisons reported off-site evaluation of acute pregnancy problems with no change during COVID
On site high-risk prenatal consultations	6 (50)	3 (75)	0	0	One jail and six prisons reported off-site high-risk prenatal consultations with no change during COVID
Telehealth	11 (100)	3 (75)	4 (36)	2 (67)	Six sites that changed all increased use of telehealth for routine medical, prenatal, and/or specialist appointments.
Changes to “Sick call” (clinic visits for acute symptoms)	N/A	N/A	3 (30)	1 (25)	One site initiated internal telehealth. One site indicated that medical staff began to respond to sick calls directly in housing units rather than in the site's clinic.
Co-pay charges for sick calls	5 (56)	4 (100)	3 (33)	1 (25)	Co-pay charges waived due to COVID
Able to access abortion in custody	10 (77)	2 (67)	0	0	One jail did not provide access to abortions before nor during COVID, and another jail reported they meet state and federal guidelines both before and during COVID Three prisons did not provide abortions before nor during COVID, and another prison reported they are unsure about abortion access during COVID, but did allow access before COVID
Labor inductions scheduled routinely at 39 weeks	5 (36)	0	0	0	
Doula/birth companion services	5 (100)	0	1 (20)	0	One site reported that prenatal doula visits and Lamaze education changed from on-site to virtual conferencing; delivering hospital restricted visitation during COVID, but this could still include a doula for an incarcerated patient.
Parenting classes	7 (100)	2 (50)	3 (43)	2 (100)	Two sites changed parenting classes to be virtual Three sites stopped all programming and visits, with no indication of virtual alternatives.
On-site mother-infant care program (“nursery program”)	4 (40)	0	0	0	
Contact visits with newborns	7 (100)	0	4 (57)	0	Four sites reported that all forms of in-person visitation ceased
Breast milk expression/storage	4 (50)	1 (25)	0	0	
Infant placement assistance (e.g., case management for adoption, foster care)	4 (50)	0	0	0	
Changes to “Sick call” (clinic visits for acute symptoms)	N/A	N/A	3 (30)	1 (25)	One site initiated internal telehealth. One site indicated that medical staff began to respond to sick calls directly in housing units rather than in the site's clinic.
Telehealth	11 (100)	3 (75)	4 (36)	2 (67)	Six sites that changed all increased use of telehealth for routine medical, prenatal, and/or specialist appointments.
Co-pay charges for sick calls	5 (56)	4 (100)	3 (33)	1 (25)	Co-pay charges waived due to COVID

^a^ Some responses had missing data. Proportions were calculated based on the number who responded to each question, according to prison or jail.

^b^ Proportions of those that had a change during COVID were calculated based on the number who provided the service pre-COVID, according to prison or jail.

N/A, not applicable.

Sites reported a variety of circumstances for quarantining pregnant people (Table 3), with reported duration from 10 to 14 days. Most prisons quarantined people on arrival to the facility, and nearly half reported quarantining pregnant people after off-site medical visits and/or after hospitalization for birth. Two prisons that provided medications for opioid use disorder in pregnancy through off-site medication provision also placed pregnant people in quarantine after transport for dosing. Four sites indicated that pregnant people needing quarantine were held in a designated quarantine unit or medical holding cell. Two sites (one jail, one prison) reported using “SHU [special housing unit]” or “restrictive housing unit,” terms used to describe solitary confinement arrangements, for quarantining pregnant people. In terms of depopulation efforts, several prisons and jails reported prioritizing pregnant people for early release (Table 3).

**Table 3.** COVID-Specific Quarantine and Confinement Practices for Pregnant, Incarcerated People

Practice	Prisons^a^ (***n***=13)	Jails^a^ (***n***=4)	Details
Quarantine			
Quarantine pregnant people on arrival to facility	10 (77)	1 (25)	
Quarantine pregnant people after off-site medical visit	6 (46)	0	
Quarantine pregnant people after hospitalization for childbirth	6 (46)	0	
Accommodations during quarantine			
Recreation and outdoor time >1 h/day	7 (70)	2 (50)	
Access to phone calls	9 (90)	2 (50)	
Access to personal property	8 (80)	3 (75)	
Commissary	8 (80)	2 (50)	
Depopulation practices			
Prioritized early release of pregnant people	3 (30)	2 (50)	Five sites reported that varying authorities called for the early release of pregnant people from their facilities during COVID One site (not counted as a change) indicated that they have had no pregnant people come into their facility since the pandemic started but did not indicate if this was due to policy change.,
Changes to arrest and detention of pre-trial people	N/A	4 (100)	All jails reported that varying authorities called for diversion or early release of high-risk populations, and other efforts such as including ticketing misdemeanor charges instead of arresting, declining to detain other charges such as “low-level marijuana cases.” One jail reported that their overall population was 50% lower than pre-COVID levels.
Changes to arrest and detention for parole or probation violation	2 (22)	0	One site elaborated that “Nobody is being released prior to their expected release date (ERD), but once they reach their ERD they are released unless they are positive for COVID - they remain until they have two negative test results.” The same site reported that limited transfers into prison were being conducted during COVID.

^a^ Some responses had missing data. Proportions were calculated based on the number who responded to each question, according to prison or jail.

ERD, expected release date.

## Discussion

Data indicate that, in response to COVID-19, modifications to prenatal care, support programming, housing practices, and release practices for pregnant people were highly variable. Most sites reported efforts to continue access to prenatal care, sometimes by expanding telehealth or increasing off-site arrangements. Many of the quarantine practices used for pregnant people raise concerns about the frequency and conditions of quarantine. Most institutions suspended in-person pregnancy support programs and newborn visits, some with no virtual alternative. We also found variability in whether pregnant people were prioritized for depopulation efforts for infection control.

This variability in COVID changes is not surprising, given that the availability and comprehensiveness of pregnancy care already varied tremendously among carceral institutions pre-COVID.^12–14^ Some study findings, such as increased use of telehealth, suspension of in-person visitation, and quarantine upon arrival to the facility, have been reported as COVID-related, nonpregnancy-specific changes at other prisons.^15,16^ Our study highlights how these and other changes play out specifically for the unique needs of pregnant people in custody.

While infection control may require housing rearrangement, it is concerning that two facilities used solitary confinement-type arrangements for quarantine, especially given dangers of solitary confinement for pregnant people, including psychological harm and lack of timely access to medical care.^17,18^ Such practices are also out of line with recommendations for the care of this population during COVID from the American College of Obstetricians and Gynecologists.^19^ Quarantine following off-site appointments may expose pregnant people to constant restrictive housing, given the frequency of prenatal visits. Furthermore, such quarantine requirements after off-site visits highlight a double standard, as they do not apply to custody officers who accompany pregnant individuals off-site, and who also go in and out of the facility each day.

Access to in-person visitation and support programs coincided with the suspension of these programs nationally, regardless of pregnancy status. However, it was troubling that most had no virtual alternative for infant and support program visits, as this may exacerbate feelings of isolation, already common among incarcerated pregnant and postpartum people.^20^

Study limitations include a small sample size, selection bias, and desirability bias. As a study conducted in the first year of the pandemic, we could not assess changes as COVID-19 continues; relatedly, we did not assess vaccine access and the re-entry process for pregnant, incarcerated individuals. Neither were we able to capture reports from pregnant incarcerated people with lived experience. Furthermore, baseline policies do not encompass the actual behaviors by facility staff. Despite this, our study contributes important policy data to a population that has been overlooked.

## Conclusions

This study demonstrates that prisons and jails made changes to their policies and practices because of COVID, some of which could have negative long-term health implications for incarcerated pregnant people. This study should serve as a catalyst for research about COVID-19 impacts on pregnant and postpartum incarcerated populations, as well as draw attention to the need for the standardization and oversight of pregnancy care in U.S. prisons and jails.

Measures to protect the well-being of pregnant incarcerated people include acknowledging that pregnant people should be prioritized in prison and jail depopulation efforts, including early release, diverting those with low-level charges for community supervision, and avoiding arrest.^19^ Other responses should ensure continued access to routine and acute pregnancy care, potentially with telehealth, eliminating use of solitary confinement for pregnant people and developing comprehensive reentry. These feasible actions, which some have already undertaken, can promote safety and equity for this marginalized group of pregnant individuals and their infants.

## Abbreviations Used

DOC: Department of Corrections
ERD: expected release date
N/A: not applicable
PIPS: Pregnancy and Prison Statistics
U.S.: United States
